# Clinical Efficacy and Safety of Lacosamide as an Adjunctive Treatment in Adults With Refractory Epilepsy

**DOI:** 10.3389/fneur.2021.712717

**Published:** 2021-12-03

**Authors:** Liyan Hou, Bingjie Peng, Defu Zhang, Jingjing Yang, Ying Wang, Li Tong, Sheng Li, Qingshan Wang, Jie Zhao

**Affiliations:** ^1^School of Public Health, Dalian Medical University, Dalian, China; ^2^Department of Biochemistry and Molecular Biology, Dalian Medical University, Dalian, China; ^3^Office of Academic Affairs, Dalian Medical University, Dalian, China; ^4^Department of Neurology, The First Affiliated Hospital of DaLian Medical University, Dalian, China; ^5^National-Local Joint Engineering Research Center for Drug-Research and Development (R&D) of Neurodegenerative Diseases, Dalian Medical University, Dalian, China

**Keywords:** antiepileptic drugs, seizures, add-on therapy, meta-analysis, randomized controlled trial, real-world study

## Abstract

**Background:** Lacosamide (LCM), a novel AED (antiepileptic drug), was used as an adjunctive treatment in patients with partial-onset seizures or without secondary generalization. However, no meta-analysis was performed to evaluate the efficacy of LCM as an adjunctive treatment in post-marketing clinical studies.

**Aims:** To assess the safety and efficacy of LCM as an adjunctive treatment in adults with refractory epilepsy, a systematic review and meta-analysis of randomized controlled trials (RCTs) and real-world studies were performed.

**Methods:** All studies were identified from electronic databases. Both RCTs and observational prospective studies were included. Primary outcomes included responder rate, adverse effects (AEs) and withdraw rate. The pooled rates (PR) with their corresponding 95% confidence intervals (CI) were calculated. Publication bias was assessed with Begg's or Egger's tests.

**Results:** Total 16 studies (3,191 patients) including 5 RCTs and 11 real-word studies were enrolled. The pooled 50% responder rate and seizure-free rate were 48% (95% CI: 0.42, 0.54) and 9% (95% CI: 0.06, 0.11) in all studies, respectively. Subgroup analysis showed that the pooled 50% responder rate were 53% (95% CI: 0.44, 0.62) from observational studies and 38% (95% CI: 0.35, 0.42) from RCTs, respectively; the pooled seizure-free rate were 13% (95% CI: 0.09, 0.18) from observational studies and 4% (95% CI: 0.06, 0.11) from RCTs, respectively. Similar incidence of AEs were reported in real-world studies (0.57, 95% CI: 0.43, 0.72) and RCTs (0.59, 95% CI: 0.42–0.76). Finally, a total of 13% (95%CI: 0.09, 0.16) and 13% (95% CI: 0.08, 0.16) of all patients prescribed with LCM was withdrawn in RCTs and real-world studies, respectively, due to the occurrence of AEs. Furthermore, similar to the 50% responder rate, seizure-free rate, incidence of AEs and withdraw rate were reported at 6-month or at least 12-month of LCM adjunction. Publication bias was not detected in these studies.

**Conclusions:** Our results revealed that LCM adjunctive therapy even with long-term treatment was efficacious and well tolerated in adults with refractory epilepsy.

## Introduction

Epilepsy is one of the most common neurological disorders with high prevalence and affects approximately 0.5–1% of the general population worldwide (1). Although many patients with epilepsy are able to achieve seizure control with anti-epileptic drugs (AEDs) treatment, 20–30% of these patients not only continue to suffer from ongoing seizures and but also experience adverse effects from the treatment (2). Patients who are unable to obtain satisfactory seizure remission on two or more different AEDs therapies are usually referred as having refractory epilepsy (3). Studies have shown that refractory epilepsy is associated with increased morbidity and mortality, serious psychosocial consequences, social impairments, which limit employment and also decrease life quality (4). Despite of rapid development of new therapeutic strategies, AEDs still play important roles in managing epilepsy in clinic.

Lacosamide (LCM), a novel antiseizure medication, was approved in 2008 by US FDA and European Medicines Agency (EMA) as an adjunctive treatment in adults with partial-onset seizures or without secondary generalization (5). In contrast to classical sodium channel-blocking AEDs that act preferentially on the fast inactivation component by shifting the voltage dependence of inactivation to more hyperpolarized potentials, LCM selectively enhances slow inactivation of voltage-gated sodium channels through binding to the collapsin response mediator protein 2 (6, 7). In 2013, a meta-analysis of randomized controlled trials (RCTs) showed that LCM appears to be a safe, efficacious and cost-effective adjunctive therapy for partial-onset epileptic seizures in adults (8). Recently, Babar and colleagues also evaluated the efficacy and tolerability of LCM as an add-on therapy for children and adults with drug-resistant focal epilepsy (9). However, previous two studies only included RCTs and the trial duration ranged from 24–28 weeks, the longer-term efficacy (LCM treatment ≥6 months) remains unknown. Although RCTs are the gold standard for evaluation of antiseizure medication treatment, there is a critical role for observational studies in extending what we learn from initial trials. In additional to confirming the consistency of efficacy and safety of antiseizure medication treatment when applied to routine practice, observational studies in the real-life clinical practice can ascertain treatment patterns (adoption, dosing, AEs, and so on) and gather data from populations not included in the RCTs (e.g., brain tumor-related epilepsy, nocturnal seizures). Recent observational studies reporting on post-marking clinical experience with LCM provide supplementary information revealed both benefits and adverse effects (AEs) of LCM as an adjunctive treatment in patients with refractory epilepsy in day-to-day clinical practice (10, 11). To date, no meta-analysis was performed to evaluate the efficacy of LCM as an adjunctive treatment in real-world observational studies that has played important roles in exploring biological efficacy of therapeutic intervention (12). Therefore, the efficacy, safety and tolerability of LCM should be further validated, especially in refractory epilepsy by enrolling both RCTs and real-world observational studies with different time-period treatment.

The present study is therefore designed to carry out a comprehensive meta-analysis to assess the safety, efficacy and AEs of LCM as an adjunctive treatment for patients with refractory epilepsy by summarizing current evidences derived from updated RCTs and high-quality observational studies with different time-period treatment, providing useful information for developers and prescribers in routine clinical practice.

## Methods

### Systematic Literature Search

Our meta-analysis adhered to the Preferred Reporting Items for Systematic Reviews and Meta-analyses (PRISMA) principles (13). All relevant articles identified through electronic searching of PubMed, Embase, Chinese National Knowledge Infrastructure, and Wanfang (China) databases up to May 18, 2020. The following search strategy was used: (drug-resistant epilepsy OR refractory epilepsy OR uncontrolled seizure OR Focal epilepsy OR generalized epilepsy OR partial-onset seizures) AND (Lacosamide) in title/abstract. The subjects of studies were defined as human, and the languages of articles were limited to English and Chinese because the reviewers are fluent in both languages. If more than one article were published using the same data, only the study with largest sample size was included. Additionally, a manual search was also conducted to retrieve additional literature from the reference lists of relevant review article.

### Inclusion and Exclusion Criteria

Selection criteria of RCTs or observational studies included in our meta-analysis were: 1) subjects confirmed to adults (age ≥16 years); 2) adult participants with focal seizures are unable to obtain satisfactory seizure remission at least 2 AEDs according to the guideline of International League Against Epilepsy (ILAE); 3) provision of at least two outcomes of interest from 50% reduction in seizure frequency comparing to baseline, seizure-free rate and side effects; 4) the treatment duration (excluding titration) was more than 8-weeks, a time that is considered to represent the minimum period to differentiate change in seizure frequency; 5) written in English or Chinese with full text available; 6) sample size more than 10. The following studies were excluded: 1) animal-based studies; 2) subjects were children and adolescents (age <16 years); 3) studies written in a language other than English or Chinese; 4) studies without original data such as comments, letters, reviews.

### Date Extraction and Outcome Measures

Two authors (Bingjie P and Li T) independently assessed the studies according to the inclusion/exclusion criteria and any discrepancies were resolved by discussion with senior authors (Qingshan W). Data collection was performed by two independent investigators (Jingjing Y and Ying W). The extracted information included the first author, publication year, number of participants, number of patients (number of patients in each group for RCTs), patients' demographic characteristics, duration of the follow-up, dosage of LCM, and outcome data.

In this meta-analysis, two clinical efficacy outcomes were assessed: 1) 50% responder rate (responders were defined as those who experienced a 50% or greater reduction in seizure frequency in the treatment period compared with baseline period); 2) seizure-free rate (seizure freedom was defined as proportion of patients that were seizure-free during treatment and follow-up period).

Two clinical safety outcomes were assessed in this meta-analysis: 1) AEs (proportion of patients experiencing any of the common AEs, such as ataxia, dizziness, fatigue, headache, nausea, and somnolence); 2) withdrawal rate due to AEs (proportion of patients with treatment withdrawal due to adverse effects of LCM).

### Date Synthesis and Analysis

The quantitative meta-analysis was performed using STATA version 12.0 (StataCorp LP, College Station, TX). The pooled rates (RRs) with their corresponding 95% CIs were calculated to assess the clinical efficacy and safety outcomes. Subgroup analyses were performed based on type of study (RCTs and observational studies) to assess the safety and efficacy of LCM as an adjunctive treatment in adults with refractory epilepsy. Considering long-term adjunctive LCM treatment (at least 12 months treatment) might cause heterogeneity, subgroup analyses were performed in observed studies. For both analysis models, the between-study heterogeneity was assessed using the Cochran's Q and I^2^ statistics (14), and a value of *I*^2^ > 50% was considered significant heterogeneity (15, 16). A random-effects model was used to calculate pooled RR in the presence (*P* ≤ 0.10) of heterogeneity (16); otherwise, the fixed-effects model was used. Begg's and Egger's tests were also applied to quantify potential publication bias, and a value of *P* < 0.05 was considered statistically significant (15).

## Results

### Literature Search

**Figure 1:**
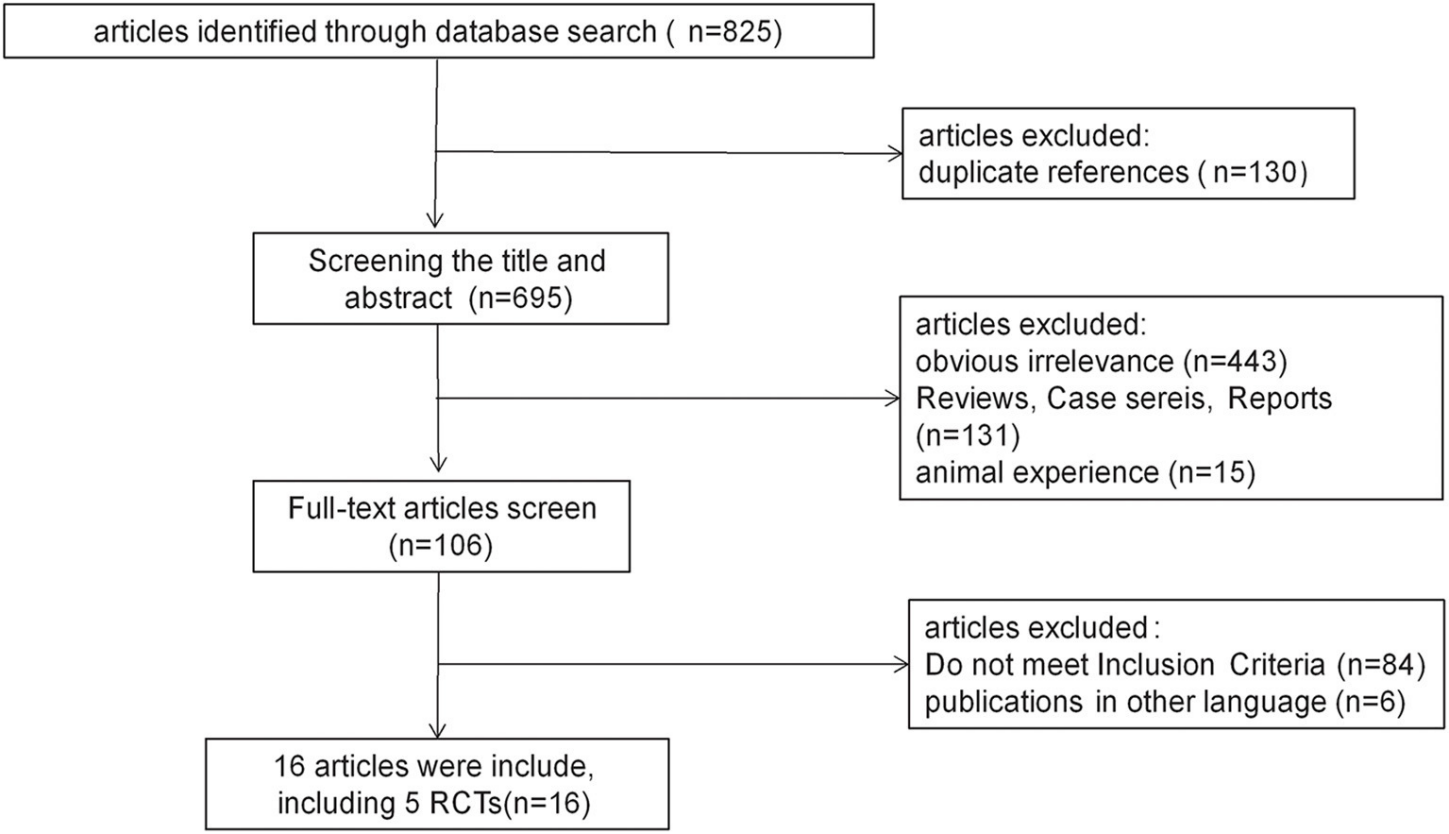
Flow diagram of study selection process.

A total of 825 relevant studies were identified. After removing duplicates by endnote software, 695 citations were independently undergone abstract review and 106 of them were considered potentially relevant trials. Among the 106 full-text articles, eighty-four studies did not meet the inclusion criteria since they were conducted on children, healthy volunteers, or indications other than epilepsy. Six studies published in other languages were also excluded. Finally, 16 studies met the inclusion criteria and were enrolled. A diagram summarizing the process of study selection is shown in Figure 1.

### Characteristics of the Enrolled Studies

The total of 16 studies enrolled in this meta-analysis included 5 RCTs (one paper published in Chinese) and 11 real-world observational studies (10, 11, 17–30). The baseline characteristics of RCTs were summarized in Table 1. All studies were published from 2007 to 2016. The age of patients was restricted to over 16 years in four studies and over 18 years in one study. All enrolled patients have had partial-onset seizures for at least the last 2 years despite prior therapy with at least 2 AEDs. In the 5 RCTs, 1,642 patients received LCM as an adjunctive treatment for refractory epilepsy. LCM at dosage of 200, 400, or 600 mg/day was administered with fixed titration schedule. All trials had a 12-week maintenance period and a 4–6 week titration period.

**Table 1:** Characteristics of the randomized controlled trials included in the present meta-analysis.

**References**	**Study design**	**Number and type of concomitant AEDs**	**Gender (male/female)**	**Age, years (mean ± SD)**	**Duration of epilepsy (Year/Month ± SD)**	**Duration of treatment**	**LCM dosage (mg/day) or Placebo**	**Seizure classification, n (%) patients**		
								**Simple partial seizure**	**Complex partial seizure**	**Secondary partial attack**
Ben-Menachem et al. (17)	8w-baseline 6w-titration period 12w-maintenance period	1-2/ CBZ, LTG, LEV, OXC, et al.	47/50 46/61 53/55 45/61	38.9 ± 11.11 39.9 ± 11.71 41.2 ± 11.61 39.4 ± 10.53	24.6 ± 11.77 25.1 ± 12.89 24.7 ± 13.08 23.6 ± 12.74	26w	Placebo 200mg 400 mg 600 mg	33(34) 48(45) 41(38) 50(47)	83(86) 101(94) 94(87) 96(91)	73(75) 79(74) 77(71) 70(66)
Halasz et al. (19)	8w-baseline 4w-titration period 12w-maintenance period 2w-transition period	1-3/ CBZ, LEV, VPA,; TPM, et al.	91/72 90/73 69/90	38.5 ± 10.93 36.9 ± 11.70 37.9 ± 12.96	21.1 ± 12.23 22.9 ± 12.30 22.8 ± 13.15	26w	Placebo 200 mg 400 mg	61(37.4) 67(41.1) 58(36.5)	138(84.7) 142(87.1) 146(91.8)	130(79.8) 125(76.7) 127(79.9)
Chung et al. (20)	8w-baseline 6w-titration period 12w-maintenance period 2w- transition period	1-3/LEV, CBZ, LGT, et al.	49/55 104/100 47/50	38.1 ± 11.96 39.1 ± 12.37 36.8 ± 11.76	25.4 ± 13.34 24.5 ± 13.16 23.4 ± 13.28	28w	Placebo 400 mg 600 mg	41(39.4) 73(36.3) 35(36.1)	86(82.7) 170(84.6) 75(77.3)	45(43.3) 84(41.8) 47(48.5)
Hong et al. (18)	8w-baseline 4w-titration period 12w-maintenance period 2w- transition period or 3w-taper period	1-3/CBZ, VPA, OXC, et al.	102/82 94/89 104/76	31.8 ± 12.0 33.2 ± 12.2 32.3 ± 11.9	16.8 ± 11.5 18.3 ± 10.9 17.9 ± 11.7	27w	Placebo 200 mg 400 mg	60(32.8) 64(35.2) 61(34.1)	183(99.5) 169(92.3) 173(96.1)	129(70.5) 114(62.6) 107(59.8)
Chung et al. (21)	4w-increasing period 12w-maintenance period 2w-reduction period	1-4/ CBZ, LEV, OXC, TMP, et al.	90/79 97/75 93/81	30.54 ± 12.04 29.61 ± 12.74 30.50 ± 11.18	158.7 ± 110.57m 159.6 ± 101.17m 172.9 ± 120.60m	18w	Placebo 200 mg 400 mg	33(19.53) 36(20.93) 38(21.84)	105(62.13) 102(59.30) 104(59.77)	72(42.60) 68(39.53) 76(43.68)

*AEDs, anti-epileptic drugs; CBZ, Carbamazepine; VPA, valproic acid; LEV, levetiracetam; LTG, Lamotrigine; OXC, Oxcarbazepine; TPM, Topiramate; SD, standard deviation*.

The main characteristics of observational studies were summarized in Table 2. Eleven real-world observational studies published from 2012 to 2018 were included. A total 1,549 patients with LCM as an adjunctive treatment for refractory epilepsy were enrolled. In addition to LCM, all patients were treated with one to three other AEDs. The duration of LCM treatment was ranged from 6 to 24 months. Since the data in one study were provided by “first add-on” and “later add-on” cohort, we analyzed the data separately and considered them to be separate studies.

**Table 2:** Characteristics of the observational studies included in this meta-analysis.

**References**	**Number of patients**	**Study design**	**Type of concomitant AEDs**	**Gender** **(male/female)**	**Age[mean ± SD or mean (range)]**	**Time since diagnosis, years (mean ± SD)**	**Duration of follow-up (Months)**	**LCM dosage (mg/day)**	**Seizure classification, number of patients**			
									**A**	**B**	**C**	**D**
Kleist et al. (29)	80	Add-on	VPA, LEV, LTG, et al.	51/29	36.2 ± 12.8	27.9 ± 13.9	24 months	300–400 mg	**patients with intellectual disability**			
Zadeh et al. (26)	456	Frist add-on Later add-on	VPA, CBZ, OXC, et al. VPA, CBZ, OXC, et al.	53/43 180/180	41 ± 17.08 38 ± 12.34	1.1 ± 2.22 22.9 ± 13.11	24 months	300–400 mg 300–400 mg;	SPS(29) SPS(112)	CPS(57) CPS(259)	sGS(69) sGS(241)	- GS:(3)
García-Morales et al. (30)	60	Add-on	LEV, CBZ, LTG, et al.	28/32	38.3	27.2	24 months	200–500 mg	NS(17)	DS(43)		
Wehner et al. (24)	25	Add-on	LTG, LEV, CBZ and ZNS	12/13	16–74	NA	6 months	400 mg	Putative etiology of focal epilepsy			
Rocamora et al. (11)	49	Add-on	LEV	24/25	39.5 ± 15.5	17.1 ± 14.6	6 months	200–400 mg	SPS(20)	CPS(34)	sGTCS(23)	-
Flores et al. (27)	285	Add-on	CBZ, LEV, et al.	199/204	41.(17–82)	NA -	Mean 11.6 months	25–700 mg	LCE(39)	SGE(7)	SPE(263)	UC(11)
McGinty et al. (10)	100	Add-on	LEV, VPA, CBZ, et al.	51/49	18–84	-NA	24 months	50–300 mg	GGE(7)	LRE76()	SGE(11)	UC(6)
Maschio et al. (23)	25	Add-on	LEV	18/7	22–74	NA	6 months	100–400 mg	SPS(9)	CPS(8)	sGS(8)	-
Stephen et al. (22)	113	Add-on	CBZ, LTG, OXC, et al.	57/56	18–74	4	6 months	200–400mg	POS			
Husain et al. (28)	309	Add-on	CBZ, OXC, LEV, et al.	162/146	38.±12.46	23.8 ± 12.97	12 months	100–600mg	**POS**			
IJff et al. (25)	33	Add-on	-	9/24	37 ± 14.5	NA	Mean 7 months	100–600mg	Cryptogenic(14)	Symptomatic(19)	-	-

*AEDs, anti-epileptic drugs; SD, standard deviation; NS (the nocturnal seizure group); NA, not available; SPS, simple partial seizure; CPS, complex partial seizures; sGS, secondarily generalized seizures; GS, generalized seizures; NS, nocturnal seizure; DS, diurnal seizure; POS, partial-onset seizures; sGTCS, secondarily generalized tonic–clonic seizures; GGE, genetic generalized epilepsy; LRE, localization-related epilepsy (focal epilepsy); SGE, symptomatic generalized epilepsy; IGE, idiopathic generalized epilepsy; UC, unclassified; SPE, symptomatic generalized epilepsy; ID, intellectual disability. Abbreviation of concomitant drugs: VPA, valproic acid; LEV, levetiracetam; CBZ, Carbamazepine; LTG, Lamotrigine; OXC, Oxcarbazepine, TPM, Topiramate; ZNS, Zonisamide. add-on: The ‘first add-on' cohort of patients received lacosamide as their first adjunctive treatment after a first monotherapy, while the ‘later add-on' cohort had previously been treated with at least two prior AED treatment regimens before adding lacosamide*.

### Clinical Efficacy Outcomes Meta-Analysis

**Figure 2:**
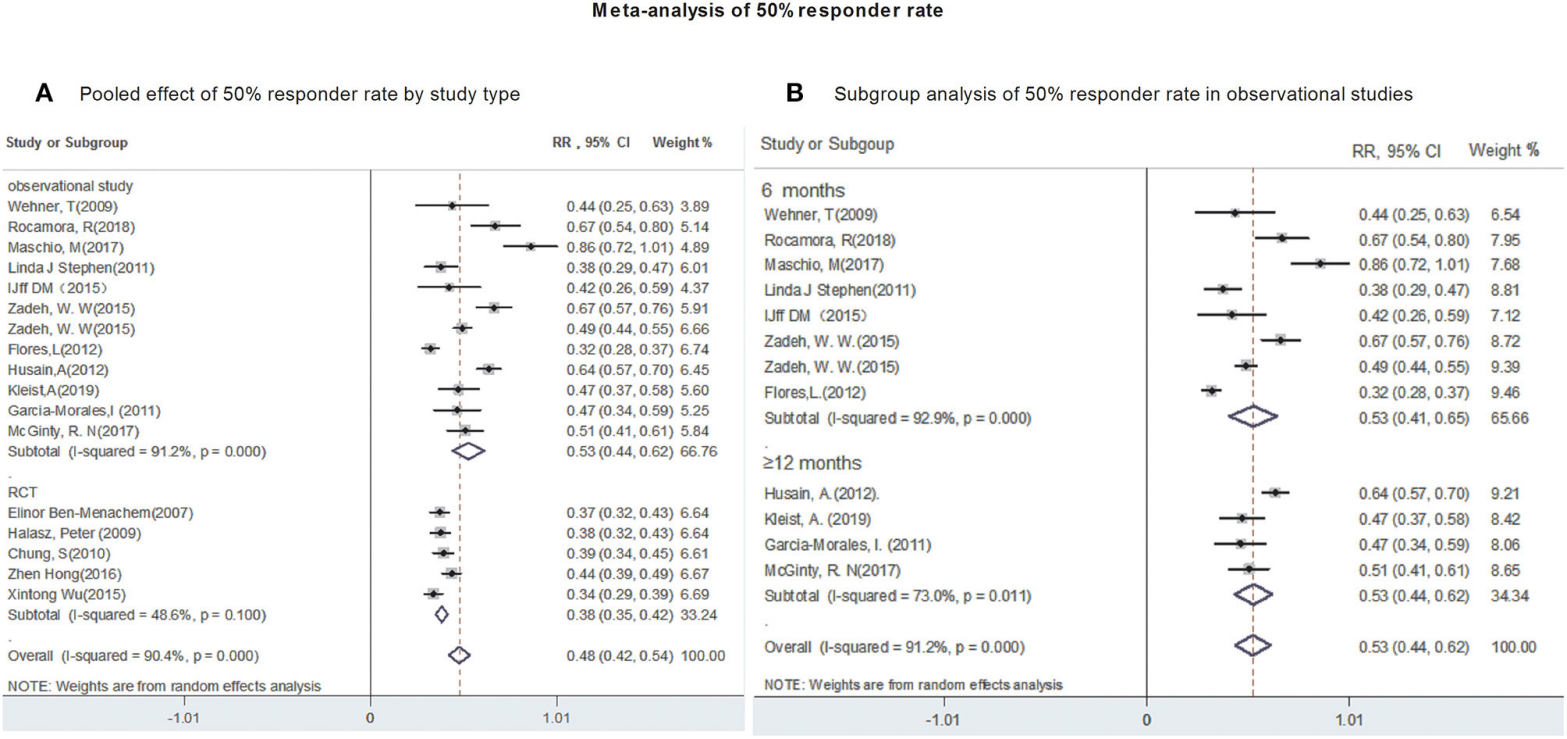
Meta-analysis of 50% responder rate. **(A)** Poole effect of 50% responder rate by study type. **(B)** Subgroup analysis of 50% responder rate in observational studies.

**Figure 3:**
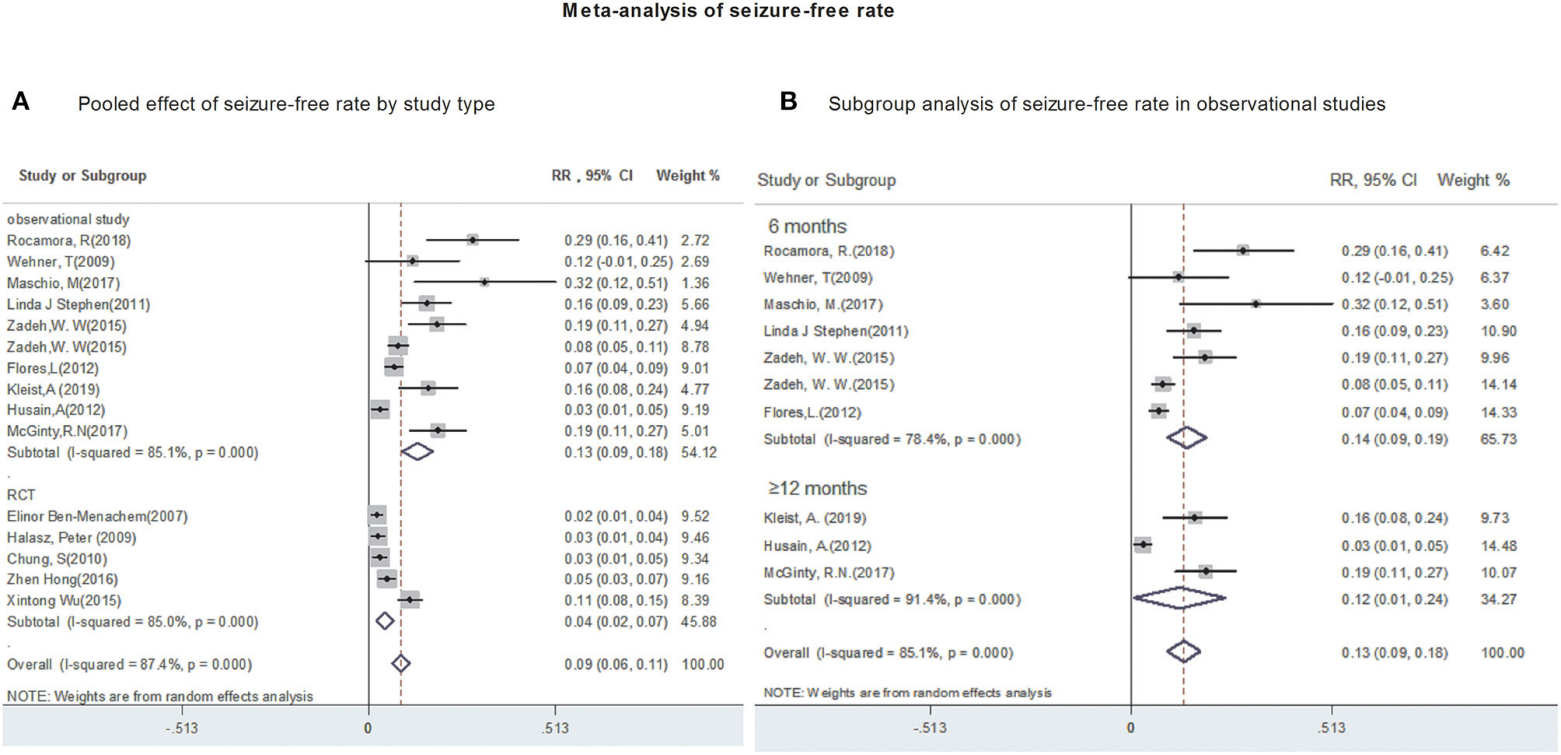
Meta-analysis of seizure-free rate. **(A)** Poole effect of seizure-free rate by study type. **(B)** Subgroup analysis of seizure-free rate in observational studies.

All studies provided data regarding seizure-frequency reduction from baseline ≥50% in response to LCM adjunction. Due to substantial heterogeneity (heterogeneity: *P* = 0.00, *I*^2^ = 90.4%), a random-effects model was used to calculate pooled RR and corresponding 95% CI. The pooled 50% responder rate was 48% (95% CI: 0.42, 0.54) (Figure 2A) in all studies. Our subgroup analysis showed that the pooled 50% responder rate were 53% (95% CI: 0.44, 0.62) from observational studies and 38% (95% CI: 0.35, 0.42) from RCTs, respectively. Since the data on 50% responder rate were provided at different time points (6- & 12-month after LCM adjunction) in observational studies, they were further analyzed separately. The 50% responder rates after 6-month of LCM adjunction, ranging from 0.32 to 0.86, was available for analysis in 1,101 patients. The pooled 50% responder rate after 6-month of LCM adjunction was 53% (95%CI: 0.41, 0.65; *P* = 0.000) (Figure 2B) with high heterogeneity (*I*^2^ = 92.9%, *P* = 0.000). Furthermore, the 50% responder rates for long-term (at least 12 months) of LCM adjunction was analyzed from the date provided by 448 patients in 5 observational studies and a rangement from 0.47 to 0.64 was observed. The pooled 50% responder rate for long-term of LCM adjunction was 53% (95%CI: 0.44, 0.62; *P* = 0.000) with high heterogeneity (*I*^2^ = 91.2%, *P* = 0.000) (Figure 2B). No publication bias was seen based on Begg's (*p* = 0.06). Fifteen studies provided data of seizure-free rate in response to LCM adjunction. The overall pooled seizure-free rate was 9% (95% CI: 0.06, 0.11) (Figure 3A) with high heterogeneity (*I*^2^ = 87.4%, *P* = 0.000). Our subgroup analysis showed that the pooled seizure-free rate were 13% (95% CI: 0.09, 0.18) from observational studies and 4% (95% CI: 0.06, 0.11) from RCTs, respectively. Nine observational studies provided data of seizure-free rate in response to LCM adjunction. The data regarding seizure-free rate (ranged from 0.07 to 0.32) at 6-month of LCM adjunction, were provided in 6 studies (including 1,068 patients). The pooled seizure-free rate at 6-month of LCM adjunction was 14% (95%CI: 0.09, 0.19; *P* = 0.000) with high heterogeneity (*I*^2^ = 78.4%, *P* = 0.000). Furthermore, the seizure-free rate (ranged from 0.03 to 0.19) for long-term of LCM adjunction, was analyzed in 3 studies including 411 patients. The pooled seizure-free rate for long-term of LCM adjunction was 12% (95%CI: 0.01, 0.24; *P* = 0.000) (Figure 3B) with high heterogeneity (*I*^2^ = 91.4%, *P* = 0.000). Begg's (*P* = 1) and Egger's tests (*P* = 0.98) revealed no significant publication bias among the included studies.

### Clinical Safety Outcomes Meta-Analysis

Twelve studies reported the occurrence of AEs, although most AEs were mild and transients. Due to a significant heterogeneity (heterogeneity *I*^2^ = 98.9%, *P* = 0.00), a random-effects model was used to calculate pooled RR and its corresponding 95% CI.

**Figure 4:**
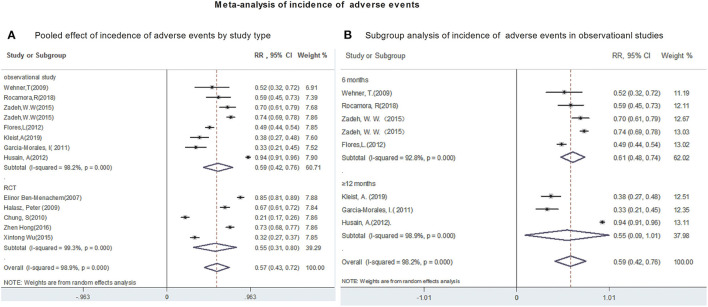
Meta-analysis of incidence of adverse events. **(A)** Poole effect of incidences of adverse effects by study type. **(B)** Subgroup analysis of incidences of adverse effects in observational studies.

The polled incidence of AEs in 12 studies was 57% (95%CI: 0.43, 0.72; *P* = 0.00) (Figure 4A). Subgroup analysis showed that the pooled incidence of AEs were 59% (95% CI: 0.42, 0.76) from observational studies and 57% (95% CI: 0.43, 0.72) from RCTs, respectively (Figure 4A). Subsequently, the incidence of AEs was analyzed in 1,441 patients after 6- and 12-month of LCM adjunction. The incidences of AEs, ranged from 0.49 to 0.73, after 6-month of LCM adjunction were provided in 3 studies including 933 patients. The pooled incidence of AEs at 6-month of LCM adjunction was 61% (95%CI: 0.48, 0.74; *P* = 0.00) with high heterogeneity (*I*^2^ = 92.8%, *P* = 0.00). Similarly, the incidence of AEs, ranged from 0.38 to 0.94, for long-term of LCM adjunction was available in 3 studies including 448 patients. The pooled incidence of AEs for long-term of LCM adjunction was 55% (95%CI: 0.42, 0.76; *P* = 0.00) with high heterogeneity (*I*^2^ = 98.9%, *P* = 0.00) (Figure 4B).

**Figure 5:**
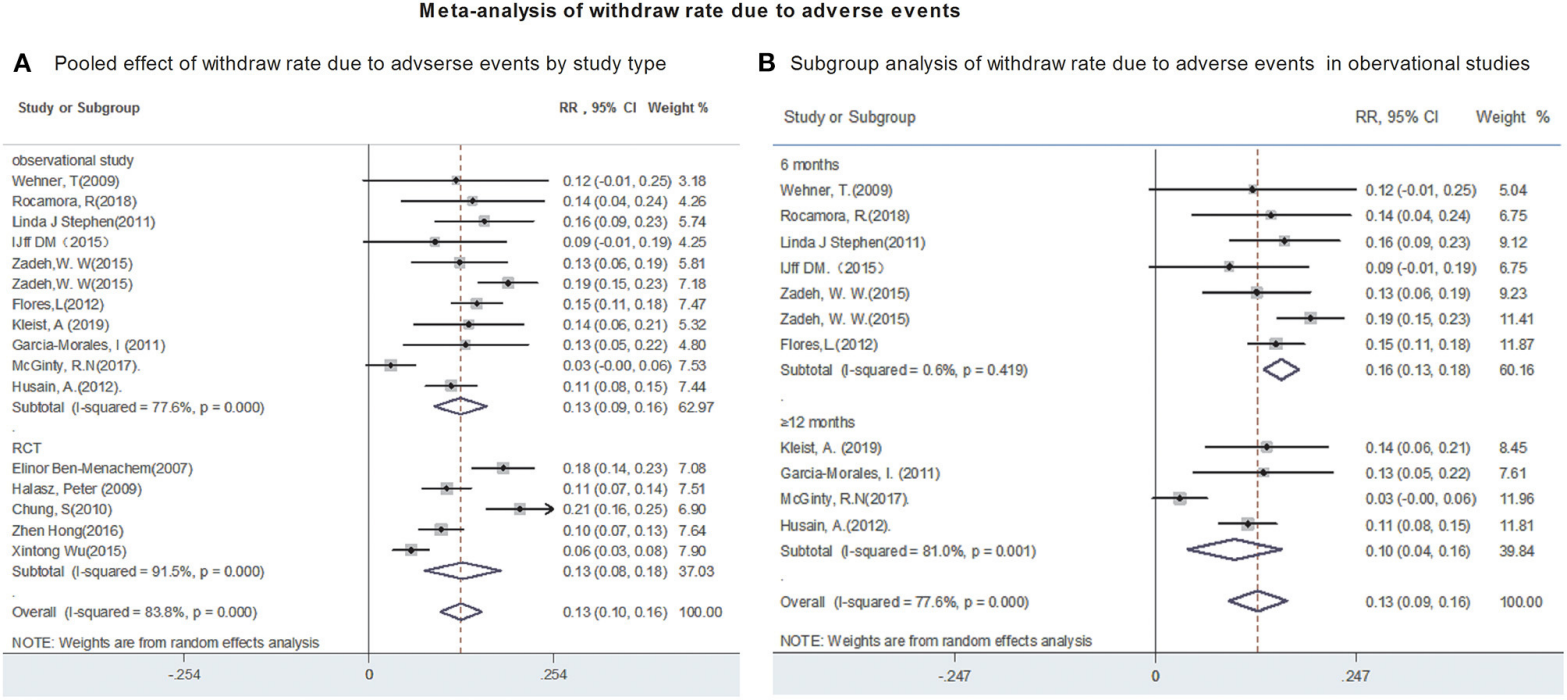
Meta-analysis of withdraw rate due to adverse events. **(A)** Poole effect of withdraw rate due to adverse effects by study type. **(B)** Subgroup analysis of withdraw rate due to adverse effects in observational studies.

Fifteen studies reported the withdraw rate due to AEs. The polled withdrawal rate in 15 studies was 13% (95% CI: 0.10, 0.16). Subgroup analysis showed that the pooled withdrawal rate due to AEs were 13% (95% CI: 0.09, 0.16) from observational studies and 13% (95% CI: 0.08, 0.18) from RCTs, respectively (Figure 5A). Further analyses revealed that the withdrawal rate after 6 and after 12-month of LCM adjunction, were 15.5% (95% CI: 0.134, 0.177) and 9.7% (95% CI: 0.039, 0.155), respectively (Figure 5B). Publication bias was not detected based on Begg's test (incidence of adverse events, *p* = 0.2; withdraw rate, *p* = 0.2).

### Discussion

To our best knowledge, this meta-analysis, for the first time, provided the comprehensive and explicit assessment of the safety and efficacy of LCM as an adjunctive treatment in adults with refractory epilepsy by using both RCTs and real-world observational studies. This study analyzed pooled data from 3,191 patients in 5 RCTs and 11 observational studies, providing practical details on how each agent licensed for developers and prescribers in daily clinical practice.

The 50% responder rate and seizure-free rate were pooled to assess the efficacy of LCM. Pooled data from 3,191 patients with LCM as an adjunctive treatment for refractory epilepsy indicated that LCM adjunction was more likely to achieve seizure control. Our findings are similar to those of previous reports, in which LCM was considered to superior to placebo in preventing seizures (8, 31). The clinical efficacy outcomes of LCM adjunction between RCTs and observational studies were further compared. The pooled result from RCTs revealed that 50% responder rate was 38%. By contrast, 53% of patients in real-world studies achieved a ≥50% reduction in seizure frequency at a short period of time (<12 months). Interestingly, the pooled 50% responder rate in real-world studies was still as high as 53% when patients treated with LCM at least 12 months, indicating that LCM appears to be effective for a long period of time. As for seizure freedom, our cumulative analysis revealed that 4% of patients became seizure-free following LCM adjunction in RCTs, whereas 13% of subjects achieved seizure freedom in real-world studies. Further, seizure freedom for a long period of time (patients treated with LCM ≥12 months) was similar to that of 6–month of LCM adjunction. Altogether, these results suggested that the efficacy outcomes of LCM adjunction for real-world studies were similar or even better than that of randomized controlled, forced titration trials. In agreement with our findings, Villanueva et al. investigated a large series of patients with partial-onset seizures treated with LCM as early add-on therapy in clinical practice and revealed a 44.9 % of seizure free rate (32). More interestingly, in Villanueva's REALLY study, good efficacy of LCM monotherapy after success polytherapy in patients with partial-onset seizures was still observed (32).

There are some factors could affect the efficacy of LCM as an adjunctive treatment in adults with refractory epilepsy. Two of the observational studies included in our meta-analysis indicated that patients were more likely to achieve seizure control when LCM was used as first add-on, compared with a later treatment schedule (22, 26). In these two studies, the 50% responder rate and seizure free rate were particularly high in patients using LCM as a first add on medication. Similar findings were observed in Rung's study, in which seizure free rate was 57.8 and 27.8%, respectively, and 50% response rate was 80.0 and 70.4%, respectively, when LCM was used in treatment as the first and later therapy (second add-on) (33). In addition, different dosage of LCM used in these two trials could also affect the efficacy and safety for seizure in adults. Patients in real-world observational studies usually used a flexible dosing regimen, while patients in RCTs were titrated to a fixed dose regiment of LCM. Moreover, the efficacy of LCM may also be affected by the treatment of other AEDs. Fifty-percent responder rate in response to LCM adjunction progressively declined with increased number of prior AEDs treatment (34). In contrast to the observational studies, patients in RCTs had tried more AEDs before LCM adjunction, which may result in decline of the efficacy.

The incidences of AEs and withdraw rate due to AEs were pooled to assess the clinical safety of LCM as an adjunctive treatment. Our results showed that the pooled incidence of AEs (including serious and non-serious AEs) was 59% in observational studies and 57% in RCTs, respectively. No significant difference between RCT and observational studies was observed. The most commonly reported AEs, which occurred in at least 10% of patients in any treatment group, focus on CNS and gastrointestinal systems (dizziness, headache, vomiting, diplopia and nausea) in RCT studies. Serious AEs, such as convulsion, dizziness, pyrexia, headache, diplopia, dysarthria, nausea, and vomiting, occurred in approximately 3–10% of all patients taking LCM in five RCT trials. Similar AEs including dizziness, diplopia, and ataxia, were also reported in observational studies. Serious AEs occurred in approximately 3–23.1% of all patients taking LCM in eleven observational studies. Although most AEs could lead to side effects (31, 35), they were nonspecific, and disappeared in some patients during the maintenance phase or with dose reduction. One observational showed that a skin rash occurred in one patient while titrating up LCM tablets, indicating an allergic reaction to a compound substance in LCM cannot be excluded (24). In addition, two patients lost more than 10% of their body weight after titration of LCM to 400 mg/day. On a group level, body weight was not affected in the RCTs that evaluated LCM. However, 2% of patients experienced a greater than 10% decrease in body weight, but the mechanism remains unclarified (36). LCM has a different mechanism of action by selectively enhancing slow sodium channel inactivation, whereas traditional sodium blockers interfere with fast inactivation pathway of sodium channel (37). The AEs in LCM group may be contributed to the pharmacodynamic interaction between LCM and other AEDs. Several studies suggested that combining drugs that block voltage-dependent sodium channels (i.e., carbamazepine, lamotrigine and valproate) are more likely to lead to side effects (22, 27). Furthermore, a total 13% of patients were withdrawn from the trial prematurely due to AEs in RCTs; while discontinuation rates were 16 and 10% for 6- and 12-month treatment, respectively in real-word studies. Our findings suggested that LCM appeared to be relatively safe and well-tolerated for a long-term treatment. Taken together, these results provided confirmatory data on the safety and safety profile of adjunctive LCM in patients with refractory epilepsy. In addition, we recommend that a reduction of the dose of concomitant traditional sodium blockers might be useful to avoid AEs (22, 29). Moreover, using a flexible dosing of LCM based on the need of each patient is recommended as an effective method to obtain satisfactory seizure control and simultaneously minimizing AEs. Clinicians should be aware that weight loss may occur.

In the past decades, the third generation antiseizure medications including brivaracetam, perampanel, eslicarbazepine acetate (ESL), cenobamate and LCM have shown efficacy to reduce seizure frequency and are fairly well-tolerated in RCTs and real-world studies (38–43). Despite direct comparison of efficacy of treatment among those third generation antiseizure medications was still lacking, Brigo's research indicated that indirect comparison meta-analyses failed to a significant difference in efficacy between add-on ESL and LCM in patients with focal epilepsy (44). Direct head-to-head clinical trials are encouraged to compare the efficacy and safety of those third generation antiseizure medications in the future.

Although this meta-analysis provided useful information for clinician to treat patients with refractory epilepsy, the following limitations should be noted. 1) Our study included 11 real-world observational studies, and some of them contained a relatively small sample size. Therefore, pooled effects using random-effects meta-analysis with a small sample size may be less precise than that of large trials. 2) There was substantial heterogeneity among the included studies. Some factors could affect the efficacy of LCM as an adjunctive treatment in adults with refractory epilepsy, such as the different dosage of LCM and concomitant AEDs, the type of epilepsy, the number of concomitant AEDs, and the duration of epilepsy. These factors have a potential impact on the pooled results. Thus, additional clinical trials with large sample size and LCM added to monotherapy are needed to further explore the potential efficacy and safety of LCM for controlling seizures.

## Conclusions

The results of the meta-analysis from RCTs and real-world observational studies confirmed that LCM is an effective and relatively safe drug when used as an adjunctive therapy in patients with refractory epilepsy. However, in many cases, patients unable to obtain satisfactory seizure remission by single AEDs are forced to take polytherapy, which could result in increase of the incidence of AEs. For these reasons, we recommend that combination of AEDs and other drugs with different mechanisms of action might be more efficacious and/or well-tolerated in patients. Otherwise, prospective reduction of concomitant sodium channels co-medications might be useful when combining with LCM to avoid AEs. Further trails are needed to assess the longer-term safety and efficacy of LCM as add-on treatment and as monotherapy.

## Data Availability Statement

The original contributions presented in the study are included in the article/supplementary material, further inquiries can be directed to the corresponding author/s.

## Author Contributions

SL and QW conceived the study and wrote the paper. BP, LT, and DZ performed the literature search. JY and YW performed data extraction. LH analyzed and interpreted data. JZ gave critical comments and revised the manuscript.

## Conflict of Interest

The authors declare that the research was conducted in the absence of any commercial or financial relationships that could be construed as a potential conflict of interest.

## Publisher's Note

All claims expressed in this article are solely those of the authors and do not necessarily represent those of their affiliated organizations, or those of the publisher, the editors and the reviewers. Any product that may be evaluated in this article, or claim that may be made by its manufacturer, is not guaranteed or endorsed by the publisher.
